# Linear streaks associated with retinitis pigmentosa

**DOI:** 10.1177/11206721241232450

**Published:** 2024-02-08

**Authors:** Maurizio Battaglia Parodi, Alessandro Arrigo, Emanuela Aragona, Adelaide Pina, Francesco Bandello

**Affiliations:** Department of Ophthalmology, IRCCS San Raffaele Scientific Institute, via Olgettina, 60, 20132, Milan, Italy

**Keywords:** Retinitis pigmentosa, linear streaks, OCT, macular neovascularization, fundus autofluorescence.

## Abstract

**Purpose:**

To describe a phenotypical manifestation characterized by the identification of peripheral linear streaks associated with retinitis pigmentosa (RP).

**Methods:**

Study design is a prospective observational case series. All consecutive patients affected by RP underwent a complete ophthalmological examination. The diagnosis of peripheral linear streaks was based on the identification of curvilinear atrophic streaks in the periphery of the retina.

**Results:**

Overall, six out of 140 patients (4.2%) were affected by peripheral linear streaks associated with RP. A single patient showed also punched out chorioretinal lesions at the posterior pole, with macular neovascularization development over the follow-up, treated with ranibizumab injections.

**Conclusions:**

RP phenotypical manifestation characterized by peripheral linear streaks is infrequent and may provide additional evidence to support the contribution of inflammation in the pathogenesis of RP.

## Introduction

The term of retinitis pigmentosa (RP) covers a heterogeneous group of diseases characterized by progressive degeneration of rod and cone cells. Clinical and functional phenotypic manifestations may significantly differ, but the key aspects are represented by difficulty in dark adaptation and night blindness, in association with a progressive loss of the visual field.^1^ RP pathogenesis is quite complex. Mutations in several genes ultimately lead to apoptotic photoreceptor death modulated by several factors. A few studies have investigated the potential role of inflammation as a modulating factor in RP.^2–6^

In our clinical activity, we have identified patients showing a phenotype combining signs typical of RP and peripheral linear streaks and designed a prospective study to investigate this association.

## Materials and methods

The study was based on a prospective observational case series. All consecutive patients affected by RP examined by the Heredodystrophy service of the Ophthalmology Department of San Raffaele Hospital from January 2016 to January 2020 were included in the study. The study was approved by the Ethical Committee of Vita-Salute San Raffaele University in Milan (NET-2016-02363765) and was in accordance with the Declaration of Helsinki. Signed informed consent was obtained from each patient before the examination. Inclusion criterion was the diagnosis of RP, which was achieved through complete ophthalmological examination, confirmed by narrowing of the visual field (30-2 Humphrey visual field) and full field electroretinogram (Utas Visual Electrodiagnostic system) with impaired scotopic response. The diagnosis of peripheral linear streaks was based on the detection of peripheral curvilinear atrophic lines, composed of multiple round chorioretinal lesions.

Exclusion criteria were as follows: any other retinal disorder (including age-related macular degeneration, degenerative myopia, Vogt-Koyanagi-Harada syndrome, multifocal choroiditis, angioid streaks, presumed ocular histoplasmosis, trauma, hereditary retinal disorders, or idiopathic macular neovascularization (MNV), tuberculosis, sarcoidosis, syphilis, birdshot retinochoroidopathy, sympathetic ophthalmia, and/or familial juvenile systemic granulomatosis); any other ocular condition that could compromise vision in the study eye.

The ophthalmologic examination included best corrected visual acuity (BCVA) using the standard Early Treatment Diabetic Retinopathy Study chart, anterior and posterior segment slit-lamp evaluation, ultrawide field color fundus photography (Optos Silverstone; Optos PLC). Optical coherence tomography (OCT) images were acquired by means of spectral domain-optical coherence tomography (Spectralis HRA + OCT, Heidelberg Engineering; Heidelberg, Germany). All patients affected by RP were invited to undergo genetic testing.

## Results

We collected data regarding 140 patients (79 males; mean age 42 ± 11 years) with clinical diagnosis of RP. All the patients were invited to undergo genetic testing, but only 78 patients (56%) attained genetic characterization. The gene most often detected was USH2A in 29 patients (∼37%), followed by ABCA4 in 18 patients (∼23%), BBS1 in 4 patients (∼5%), PROM 1 in 4 patients (∼5%), PRPF31 in 3 patients (∼4%), CYP4V2 in 3 patients (∼4%), NR2E3 in 2 patients (∼3%), PDE6A in 2 patients (∼3%), RP1L1 in 2 patients (∼3%), CNGA1 in 2 patients (∼3%), CNGB1 in 2 patients (∼3%), MYO7A in 2 patients (∼3%), EYE in 1 patient (∼1%), EYS in 1 patient (∼1%).

Overall, we identified 6 out of 140 patients (4.2%) disclosing bilateral peripheral linear streaks, 3 of them with genetic confirmation (c.165G > A variant on the PRPF31 gene, c.1169T > G variant on the BBS1 gene, and c.6118C > T variant on the ABCA4 gene).

**Figure 1.**
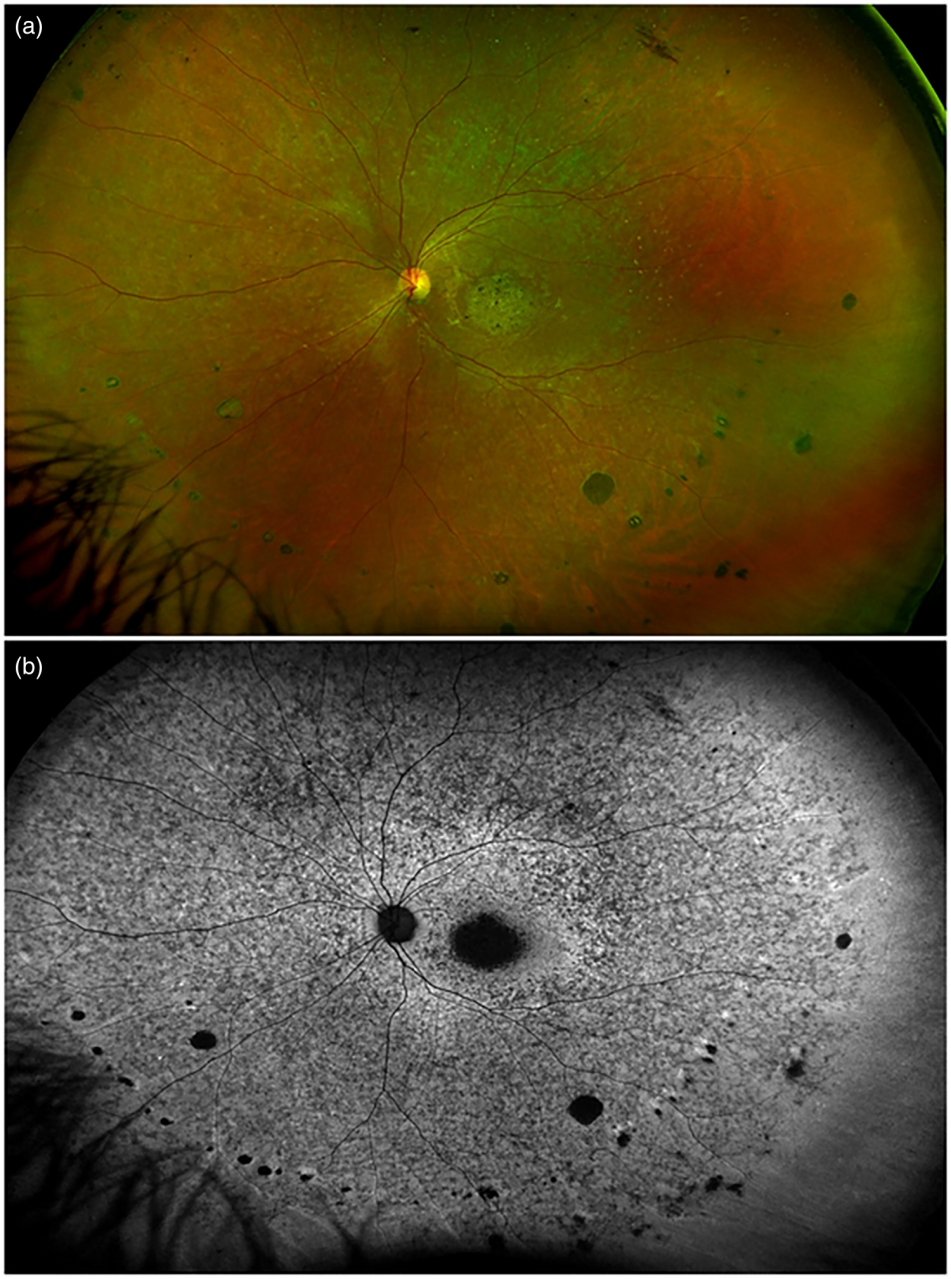
Fundus imaging in a case of retinitis pigmentosa with linear streaks. (a) Ultra-wide field color photography showing linear streaks of round chorioretinal scars in mid periphery. (b) Ultra-wide field fundus autofluorescence of the same patient disclosing the changes at the level of the retinal pigment epithelium.

The mean age of the 6 patients affected by typical RP included in the study was 34 ± 9 years, with 3 males, and a mean BCVA of 20/40. The mean follow-up was 4.1 ± 2.6 years. The anterior segment examination was unremarkable, while the biomicroscopic fundus examination revealed a variable amount of bone-spicule with atrophic changes, associated with peripheral linear streaks of small, round, punched out chorioretinal scars (Figure 1). Punched out chorioretinal lesions were identified at the posterior pole in just one case. Peripapillary atrophy was detected in 8 eyes of 4 patients. All 6 patients showed a variable number of vitreous cells over the follow-up and an elevated number of intraretinal hyperreflective foci on OCT, mainly localized between the lower part of the inner plexiform layer and the upper part of the external limiting membrane. Patient 1 displayed a small scar at the posterior pole of the left eye, patient 2 had a narrow myopic staphyloma in both eyes *(refractive error −6.00D in both eyes)*, and patient 3 was bilaterally affected by foveal atrophy. In all cases, visual field examination revealed a constriction, while the electroretinogram detected a deep defect in the rod response. Patient 1 developed a subfoveal MNV, which required 6 ranibizumab injections to be controlled, and subsequently another extrafoveal MNV, which was stabilized with 2 ranibizumab injections over the 5-year follow-up. BCVA varied from 20/80 to 20/20, according to the activity of the MNV, with a final value of 20/32.

## Discussion

Our study describes an infrequent phenotypic manifestation occurring in about 4% of cases, characterized by the typical clinical aspects of RP in combination with peripheral linear streaks. Peripheral linear streaks have been described in the clinical setting of many conditions, including multifocal choroiditis, presumed ocular histoplasmosis, degenerative myopia, Vogt-Koyanagi-Harada syndrome, angioid streaks, West Nile virus-associated chorioretinitis, and familial juvenile systemic granulomatosis.^7–12^ Moreover, linear streaks and punched-out chorioretinal scars have already been reported in association with other dystrophies, including Stargardt disease,^13^ cone dystrophy,^14^ and pseudoxanthoma elasticum.^15^

Several studies have already focused on some inflammatory alterations associated with RP,^2–6^ suggesting a contribution to the phenotypic manifestation of the disease.

Even though a simple chance association between RP and idiopathic multifocal choroiditis cannot be ruled out, we hypothesize that peripheral linear streaks may be related to an inflammatory response, which may exhibit variable clinical spectrum in RP, ranging from simple vitreous flare, to increased number of intraretinal hyperreflective foci, up to the development of chorioretinal scars.^6^ Myopic staphyloma may be also present, in this case classified as narrow type.^16^

The association of linear streaks and punched-out chorioretinal lesion features in cases of RP has direct clinical implications because vision-threatening complications such as MNV can develop in patients affected by multifocal choroiditis and other inflammatory disorders.^15^ In particular, macular scars proved to deserve closer monitoring than those outside the posterior pole, which remained stable throughout the follow up. Indeed, in our case series, case 1 developed two MNVs, leading to visual acuity deterioration and thus requiring anti-VEGF treatment.

Our study has obvious limitations and is best regarded as a conceptual aid in identifying clinical signs of inflammation complicating the course of dystrophies, such as RP. In particular, we acknowledge that since the study was based on a single center, although a relatively large number of patients were examined; furthermore, genetic characterization was limited, as almost half of the patients refused to undergo genetic testing for personal reasons. Moreover, vitreous abnormalities which are common in RP patients,^17,18^ might be implicated in the LS development. However, we did not perform dedicated analyses on the vitreous status and alterations. Further studies should be focused on the assessment of the association between LS and vitreous disorders in RP. In addition, the aim of the study was merely to investigate the association between peripheral linear streaks and RP. Other subclinical/clinical signs of inflammation would likely be detected with systematic multimodal techniques, thus providing a clearer picture of the contribution of inflammation to the pathogenesis of RP.
